# The Impact of Anti-Epileptic Drugs on Growth and Bone Metabolism

**DOI:** 10.3390/ijms17081242

**Published:** 2016-08-01

**Authors:** Hueng-Chuen Fan, Herng-Shen Lee, Kai-Ping Chang, Yi-Yen Lee, Hsin-Chuan Lai, Pi-Lien Hung, Hsiu-Fen Lee, Ching-Shiang Chi

**Affiliations:** 1 Department of Pediatrics, Tungs’ Taichung Metroharbor Hospital, Wuchi, 435 Taichung, Taiwan; fanhuengchuen@yahoo.com.tw (H.-C.F.); sagelai@yahoo.com.tw (H.-C.L.); 2 Department of Nursing, Jen-Teh Junior College of Medicine, Nursing and Management, 356 Miaoli, Taiwan; 3 Department of Pathology and Laboratory Medicine, Kaohsiung Veterans General Hospital, 813 Kaohsiung, Taiwan; herngsheng131419@gmail.com; 4 Department of Pediatrics, Taipei Veterans General Hospital, 112 Taipei, Taiwan; kaipingchang@gmail.com; 5 Division of Pediatric Neurosurgery, Neurological Institute, Taipei Veterans General Hospital, 112 Taipei, Taiwan; yylee62@gmail.com; 6 Faculty of Medicine, National Yang-Ming University, 112 Taipei, Taiwan; 7 Department of Pediatrics, Kaohsiung Chang Gung Medical Center, 833 Kaohsiung, Taiwan; flora1402@adm.cgmh.org.tw; 8 Department of Pediatrics, Taichung Veterans General Hospital, 407 Taichung, Taiwan; leehf@hotmail.com.tw

**Keywords:** epilepsy, bone metabolism, anti-epileptic drugs (AEDs), classical anti-epileptic drugs (AEDs), newer anti-epileptic drugs (AEDs), cytochrome P450 (CYP450), bone mineral density (BMD)

## Abstract

Epilepsy is a common neurological disorder worldwide and anti-epileptic drugs (AEDs) are always the first choice for treatment. However, more than 50% of patients with epilepsy who take AEDs have reported bone abnormalities. Cytochrome P450 (CYP450) isoenzymes are induced by AEDs, especially the classical AEDs, such as benzodiazepines (BZDs), carbamazepine (CBZ), phenytoin (PT), phenobarbital (PB), and valproic acid (VPA). The induction of CYP450 isoenzymes may cause vitamin D deficiency, hypocalcemia, increased fracture risks, and altered bone turnover, leading to impaired bone mineral density (BMD). Newer AEDs, such as levetiracetam (LEV), oxcarbazepine (OXC), lamotrigine (LTG), topiramate (TPM), gabapentin (GP), and vigabatrin (VB) have broader spectra, and are safer and better tolerated than the classical AEDs. The effects of AEDs on bone health are controversial. This review focuses on the impact of AEDs on growth and bone metabolism and emphasizes the need for caution and timely withdrawal of these medications to avoid serious disabilities.

## 1. Introduction

Epilepsy, a common neurological disorder, affects about 50 million people around the world. The prevalence of epilepsy is approximately 6.8 per 1000 in the US [1], 5.5 per 1000 in Europe, 1.5 to 14 per 1000 in Asia [2], and 3.3 per 1000 in Taiwan [3]. Although there are many alternative treatment choices for epilepsy, including vagus nerve stimulation (VNS), surgery, and a ketogenic diet, anti-epileptic drugs (AEDs) are always the first choice because numerous patients with epilepsy were seizure-free while taking an AED [4]. However, AEDs should be used carefully because of drug-drug interactions and potential side effects, such as dizziness, drowsiness, mental slowing, skin rashes, hepatotoxicity, movement and behavioral disorders, and metabolic disturbances, such as weight gain, metabolic acidosis, and nephrolithiasis [5]. Although few unusual adverse effects such as rickets, osteomalacia, and abnormal dentition were previously identified in patients taking the AEDs [6,7], more than 50% of patients with epilepsy who take AEDs are reported to have bone abnormalities [8,9,10], and several case-control studies have traced a link between long-term AED use, bone diseases [11,12,13], and the increase of fracture risks [12,14,15]. The newer AEDs, including levetiracetam (LEV), oxcarbazepine (OXC), lamotrigine (LTG), topiramate (TPM), gabapentin (GP), and vigabatrin (VB) are also effective in the treatment of various seizures, in addition to being safer and better-tolerated than the classical AEDs. However, studies regarding the effects of the newer AEDs on bone health and growth are limited.

Symptoms of patients with AED-associated bone diseases include bone pain, muscle weakness, and fractures, with minimal or no trauma. These symptoms do not appear until the first fracture occurs [16]. Their biochemical studies may show abnormal serum levels of vitamin D metabolites, phosphorous, Ca^2+^, and alkaline phosphatase. Routine X-rays can identify bone fractures, but cannot detect these bone diseases if the reduction of bone mass density (BMD) is less than 30% [17]. BMD represents a complex and dynamic balance between the actions of osteoclasts, which are responsible for bone resorption, and the actions of osteoblasts, which are responsible for the bone-formation. The values of BMD in twins and siblings with epilepsy receiving AEDs treatment were significantly lower than that without treatment [18]. Dual energy X-ray absorptiometry (DXA) is an X-ray technique to measure the levels of BMD [17]. One-third to two-thirds of epileptic patients with AEDs showed abnormal BMD values by using DXA [9,19], but the safety of the ionizing radiation exposure is a large concern.

Although it is clear that AEDs affect bone metabolism and increase fractures are not clear, the metabolism of drugs may play an important role in the development of these adverse effects. The metabolism of drugs can be divided into two phases. CYP 450 is responsible for the phase I metabolism, including activation, metabolism, and clearance of medications. Several medications cause unwanted side effects and decreased or no therapeutic effects because these medications, including AEDs administered parenterally or non-parenterally, can induce or suppress CYP450, leading to unanticipated drug-drug interactions [20]. Reports showed that enzyme-inducing AEDs (EIAEDs) could induce CYP450 to accelerate the degradation of vitamin D, contributing to hypocalcemia [21,22,23], reduced BMD, and a higher risk of fractures [24,25]. Studies showed that valproic acid (VPA), one of the non-enzyme-inducing AEDs (NEIAEDs), was associated with low bone mass [13,26,27]. The glucuronidation is responsible for the phase II metabolism. Organisms utilize glucuronidation to detoxify environmental toxins and carcinogens and participate in essential biochemical processes. UDP-glucuronosyltransferases (UGTs), which are the most important enzymes in the glucuronidation, comprise a superfamily of key proteins, UGT1 and UGT2. Each of the proteins UGT1 and UGT2 has at least eight isoenzymes [28]. UGTs facilitate the glucuronic acid group of uridine diphosphoglucuronic acid (UDPGlcA) transferring to several structurally diverse chemicals, such as AEDs, to increase the polarity and enhance their chemicals excretion in the urine and bile [29].

No definitive guidelines for evaluation of the effect of AEDs on bone metabolism are available. The diagnosis and the treatments of epilepsy are commonly initiated in childhood and adolescence, which are a critical period of growth in life. Therefore, it is worth conducting a short review to discuss the impact of classical and newer AEDs and how the metabolites of these AEDs affect bone health. The results of this review may allow for patients with AED-associated skeletal bone diseases to be recognized earlier and appropriate therapy to be implemented without delay.

### 1.1. Benzodiazepines (BZDs)

**Figure 1:**
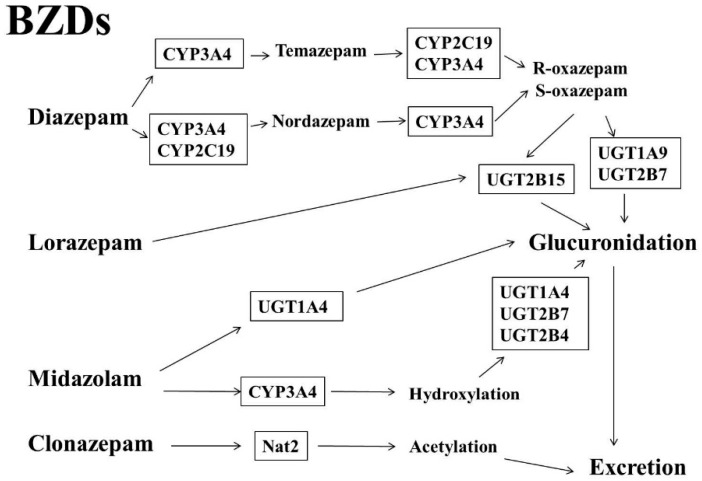
Pathways of the benzodiazepines (BZD) biotransformation. CYP: cytochrome P450; UGT: Uridine 5′-diphospho-glucuronosyltransferase.

BZDs, such as diazepam, lorazepam, midazolam, and clonazepam, are widely prescribed. Minimal toxicity and rapid onset of action make BZDs among the top 100 most commonly prescribed medications [30]. One of the main effects of BZDs is the enhancement of the neurotransmitter gamma-aminobutyric acid (GABA) and GABA receptor-mediated chloride conductance, contributing to the effects of sedation, hypnosis, anxiolysis, anti-seizure, and muscle relaxation [31,32]. Metabolism of BZDs includes liver microsomal oxidation, hydroxylation, glucuronidation, acetylation, etc. [33]. CYP3A4, CYP3A5, CYP2C19, and others are associated with the hydroxylation of BZD [34,35,36]. Some hydroxylated metabolites of BZDs still have pharmacological activities. UGTs are responsible for the process of glucuronidation of BZD [36,37]. Midazolam, S-oxazepam, and R-oxazepam undergoes glucuronidation by UGT1A4 [36,37,38], UGT2B15 [37], and UGT2B7 and UGT1A9 [37], respectively. Clonazepam undergoes acetylation by NAT2 [39,40] (Figure 1). BZD metabolites are mainly eliminated through renal excretion. A retrospective investigation concluded that the use of diazepam, lorazepam, and clonazepam [41,42] might induce a substantial number of fractures and consequential costs. Temazepam, a metabolite of diazepam via CYP3A4, was found to increase the risk of fractures [43]. There was only one case report regarding the use of oxazepam and recurrent mandibular luxation [44]. BZDs have also been reported to disturb bone metabolism, including a reduction of BMD and 25-hydroxy vitamin D (25OHD), and an increase in the serum alkaline phosphatase (ALP) levels. The levels of total calcium, phosphorus, magnesium, and parathyroid hormone (PTH) were unaffected by BZDs [24,35] although some other results are controversial [45,46,47]. Interestingly, a report showed that midazolam could exert negative effects on cell viability and osteogenic differentiation of cultured human bone marrow stem cells, suggesting a detrimental effect of the use of midazolam on bone formation and growth [48] (Table 1).

### 1.2. Carbamazepine (CBZ)

**Figure 2:**
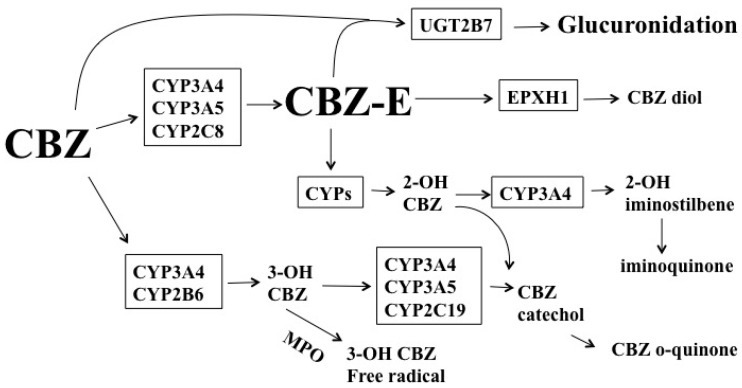
Pathways of the benzodiazepines (CBZ) biotransformation; CBZ-E: CBZ 10.11-epoxide; MPO: myeloperoxidase; EPXH1: epoxide hydrolase 1.

CBZ, an iminodibenzyl derivative, is extensively bio-transformed in the liver and approximately 5% of CBZ is eliminated through renal excretion [49]. CBZ 10.11-epoxide (CBZ-E), which possesses anti-convulsant properties, is generated through the action of CYP3A4, CYP3A5, and CYP2C8 [50,51]. CBZ diol is generated via the action of epoxide hydrolase 1 (EPXH1) (Figure 2). Although glucuronidation is not important in the metabolism of CBZ, UGT2B7 may be involved in the metabolism of CBZ and CBZ-E [52,53]. Other metabolites of CBZ include 2-OH CBZ and 3-OH CBZ. The former is generated through the actions of multiple CYPs and the latter is produced by the actions of CYP2B6 and CYP3A4 [50]. 2-OH CBZ is oxidized by CYP3A4 to produce an iminoquinone intermediate [50], whereas 3-OH-CBZ is oxidized by CYP3A4 to generate CBZ o-quinone [50]. 3-OH CBZ may generate radicals through the action of myeloperoxidase (MPO) [50].

CBZ stabilizes voltage-gated sodium channels (VGSCs), minimizes VGSCs in the rest status subsequently to be excited, and reduce polysynaptic responses to block post-tetanic potentiation. These actions make CBZ a widely used AED for partial and secondary generalized seizures [54]. Additionally, CBZ’s structure is similar to that of the tricyclic anti-depressants and a function of CBZ is a GABA receptor agonist. These may partially explain the effects of CBZ on bipolar disorder and the treatment of pain in trigeminal neuralgia [50].

CBZ may cause several adverse effects, including sedation, ataxia, dizziness, nausea, vomiting, constipation, diarrhea, interference with the metabolism of lipids and sex hormones, hyponatremia, weight-gain, anemia, agranulocytosis, toxic epidermal necrolysis (TEN), Stevens Johnson syndrome (SJS), and drug reactions with eosinophilia and systemic symptoms (DRESS) [55,56,57]. Erythromycin, clarithromycin, and triacetyloleandomycin are the most potent CYP3A4 inhibitors and are best avoided in CBZ-treated patients. Azithromycin does not interact with CYP3A4 and, therefore, does not affect CBZ concentrations.

CBZ was reported to cause spinal bifida in 1% of neonates whose mothers had an exposure history in pregnancy [58]. Moreover, long-term use of CBZ may increase the risks of fracture and bone loss, induce a status of decreased bone and mineral metabolism, increase bone turnover, and decrease BMD [19,42]. CBZ may induce CYP450 to decrease the levels of vitamin D. A study of previously drug-naive Koreans with CBZ revealed a significant decrease in BMD [59]. On the contrary, high levels of bone formation markers have been detected in patients treated with CBZ, despite normal levels of vitamin D [60]. Pack et al. [61] found that serum calcium and estrogen levels were lower in epileptic women in premenopausal status taking CBZ. However, there was no connection between bone turnover marker or calciotropic hormone levels and BMD change in these women, suggesting it was estrogen rather than vitamin D that led to bone loss in epileptic women in premenopausal status [61]. Whether CBZ affects bone through the induction of CYP450 and/or its metabolites remains unknown (Table 1).

### 1.3. Phenytoin (PT)

PT (5,5-Diphenyl-Imidazolidine-2,4-Dione) is available in oral and intravenous formulations. The bioavailability of oral PT is 70%–90% and the *t*_1/2_ of PT is 12–36 h. The peak blood level of PT is 3–12 h [62]. Adverse effects, such as nausea, vomiting, gingival hyperplasia, burning sensation at the local injection site, nystagmus, ataxia, hypotension, bradyarrhythmias, cardiac arrest, SJS, TEN, and birth defects, may occur [62]. Dissolving PT in a base solution with pH of 12 that contains sodium hydroxide, ethanol, and propylene glycol can improve the aqueous solubility. However, the cardiovascular toxicity of intravenous phenytoin infusion may contribute to the strong effects of propylene glycol on the vagal nerve [62]. Additionally, the high pH is responsible for propylene glycol’s veno-irritant properties [62]. The therapeutic range of PT is narrow and the clearance of PT is variable between individuals. Additionally, co-ingestion of PT with an antacid mixture of magnesium trisilicate and aluminum hydroxide reduces serum PT concentrations [63]. Moreover, some medications, such as Cisplatin and other anti-neoplastic drugs may affect serum PT concentrations [64]. All of them suggest that it is necessary to do therapeutic drug monitoring when using PT.

PT inhibits GABA and glutamate transport [65], reduces calcium influx into neurons to decrease the release of neurotransmitters [66], and reduces synaptic post-tetanic potentiation, and excitatory synaptic transmission to stop the cortical abnormal current propagation [67]. Moreover, PT can bind to and stabilize the inactive VGSCs [68]. VGSCs are highly conserved and responsible for the upstroke of the action potentials in neurons involving the propagation of the electrical impulse in the CNS, PNS, and cardiovascular and skeletal muscle tissue. After binding, PT prevents further generation of action potentials, which initiate seizures [68]. These mechanisms may significantly prevent generalized tonic-clonic seizures, complex partial seizures, and status epilepticus, but not absence seizures.

**Figure 3:**
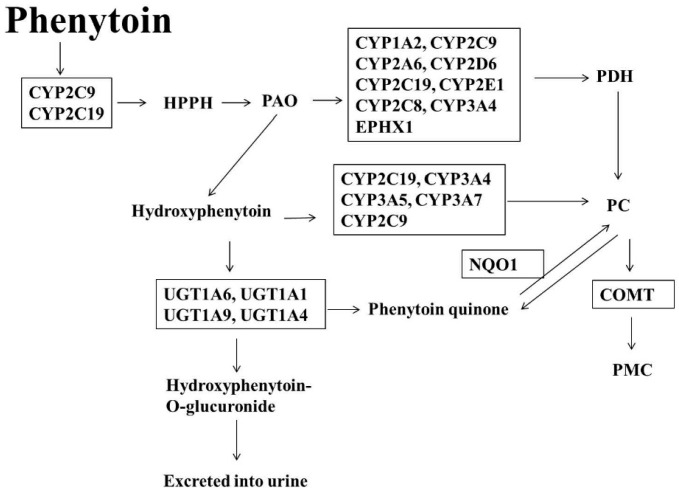
Pathways of the PT biotransformation. HPPH: hydroxyphenytoin, 5-(4′-hydroxyphenyl)-5-phenylhydantoin; PAO: phenytoin-arene oxide; PDH: phenytoin dihydrodiol; PC: phenytoin catechol; NQO1: NAD(P)H dehydrogenase, quinone 1; PMC: phenytoin methylcatechol; COMT: Catechol-*O*-methyltransferase.

PT is well-absorbed orally, and up to 90% of PT is biotransformed to HPPH, 5-(4′-hydroxyphenyl)-5-phenylhydantoin and hydroxyphenytoin [69], which are inactive metabolites and are excreted into urine after glucuronidation [70]. HPPH proceeds to form phenytoin-arene oxide (PAO), which may be the reason why epileptic patients develop hepatotoxicity, hypersensitivity, TEN, SJS, and idiosyncratic toxicity after taking PT [71]. PAO is metabolized to phenytoin dihydrodiol (PDH) via CYP1A2, CYP2C19, CYP2E1, CYP2A6, CYP2D6, CYP2C8, CYP2C9, CYP3A4, and epoxide hydrolase (EPHX1) [69,72]. Phenytoin catechol (PC) is a downstream metabolite of PDH [69]. Hydroxyphenytoin is turned into PC through the actions of CYP2C19, CYP3A4, CYP3A5, CYP3A7, and CYP2C9 [69,72,73]. PC is spontaneously and reversibly oxidized to form a phenytoin quinone by NAD(P)H dehydrogenase, quinone 1 (NQO1). PC is converted to phenytoin methylcatechol (PMC) through the action of Catechol-*O*-methyl transferase (COMT) [69]. Hydroxyphenytoin is glucuronidated by UGT1A1, UGT1A4, UGT1A6, and UGT1A9 [74]. PT can induce CYP3A, CYP2C, and UGTs [75] (Figure 3).

Fetal hydantoin syndrome is characterized by learning disabilities, low IQ scores, growth retardation, microcephaly, and facial dysmorphologies [76], suggesting a significant influence on bone growth. PT might induce a substantial number of fractures and consequential costs [42] in vivo and in vitro [77,78]. PT may also induce the expression of CYP450, which increases the degradation of bioavailable vitamin D, decreases absorption of calcium in the gut, decreases serum levels of calcium and phosphate, and increases PTH. These effects may then lead to increased bone turnover, reduced BMD, and increased susceptibility to fractures [79,80,81]. Among phenytoin’s metabolites, only HPPH was found to affect bone in vitro [82]. Therefore, the bone condition of patients taking PT should be monitored regularly (Table 1).

### 1.4. Phenobarbital (PB)

PB (5-ethyl-5-phenyl-1,3-diazinane-2,4,6-trione) was the most commonly-used AED in the world [83,84]. PB is available in oral and intravenous formulations. Its pharmacokinetics are linear and protein binding is 55%. The bioavailability of oral PB is more than 95% and the peak blood level of PB is 0.5–4 h. The *t*_1/2_ of PB is 2–7 days [85]. Discontinuing PB should be done with caution because a case report showed an increase of seizure frequency in patients tapering the doses of PB while stabilized on another AED [86]. Twenty-five percent of PB is cleared by renal excretion in unchanged form [87]. After administration, PB was detected in hepatic tissue and the portal vein, vena cava, and aorta [88], suggesting that the liver is the main organ for the metabolism of PB. The metabolites of PB include free PB and two inactive metabolites. p-hydroxy PB (6%–40% of the dose) is created by CYP2C9, CYP2C19, and CYP2E1 through the process of aromatic hydroxylation and 9-d-glucopyranosyl-PB by glucuronidation (25% of the dose). The enzymes involved in this *N*-glucosidation have not yet been identified; however, UGT 2B has been proposed as the enzyme responsible for this process [89]. These processes are complicated and exhibit a large inter-individual variability [90]. Orphan nuclear receptors, including pregnane X receptor (PXR) and constitutive androstane receptors (CAR), are activated by PB to upregulate CYP 450 gene expression [91], causing increased clearance and decreased serum concentrations of drugs, including AEDs (e.g., CBZ, PT, VPA, LTG, TPM), and lipid-soluble drugs (e.g., oral contraceptives, warfarin, corticosteroids, sex hormones, vitamin D) [92]. VPA may change serum levels or prolong the *t*_1/2_ of PB by affecting the metabolism of PB [92,93], leading to variable dose requirements for PB. Therefore, therapeutic drug monitoring of PB levels is needed when PB is used in combination with other drugs.

PB enhances GABA and GABA_A_ receptor-associated inhibition [94] and facilitates Cl^−^ conductance by extending the time of channel opening [95]. These effects lead to an increased Cl^−^ influx to hyperpolarize the postsynaptic neurons and block the propagation of aberrant epileptic currency. PB may directly activate the GABA_A_ receptor [96]. The actions of PB through these effects may reduce anxiety, promote sleep, induce general anesthesia, and act as an effective control of generalized and partial tonic–clonic seizures [97,98]. PB was the World Health Organization’s first-line AED in developing countries because of its low cost and effectiveness in the treatment of seizures. However, the use of PB has decreased even though there is no obvious connection between the use of PB and the development of behavioral problems [99].

Side effects, such as sedation, hypnosis, dizziness, nystagmus, ataxia, excitement, confusion, and paradoxical hyperactivity may occur. Contraindications for PB use include acute intermittent porphyria, hypersensitivity to PB, a prior history of dependence on PB, and hyperkinesia in children [62]. In vivo studies showed that long-term use of PB might diminish the *t*_1/2_ of the plasma vitamin D3 and enhance excretion in the bile [100]. Long-term use of PB may increase the risks of fracture [42] and bone loss [19]. Liver microsomes dissected from animals with PB treatment enabled vitamin D3, 25-hydroxycholecalciferol, and 1,25-dihydroxycholecalciferol to turn into inactive products [100], causing rickets, osteomalacia, and hypocalcemia. Therefore, vitamin D supplementation should be considered for patients receiving long-term PB therapy. p-hydroxy PB and 9-d-glucopyranosyl-PB have not been reported to be associated with bone diseases (Table 1).

### 1.5. Valproic Acid (VPA)

**Figure 4:**
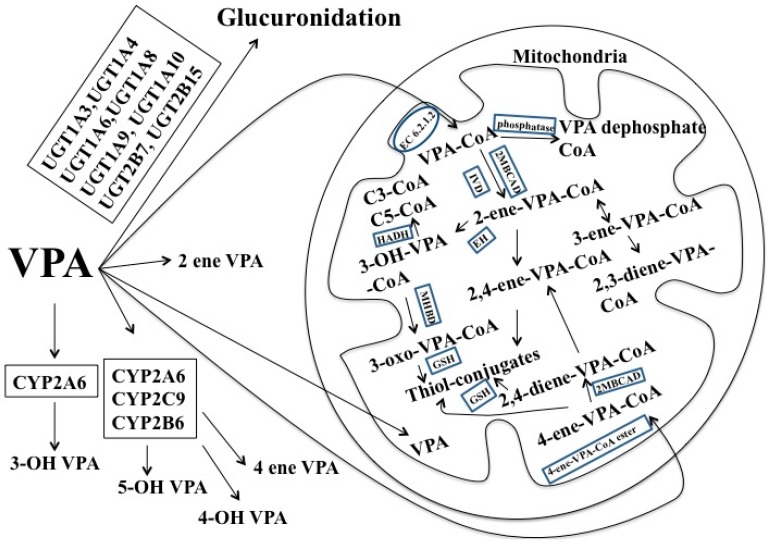
Pathways of the VPA biotransformation. VPA-CoA: valproyl-CoA; EC 6.2.1.2: medium-chain acyl-CoA synthase; 2-ene-VPA-CoA: 2-propyl-valproyl-CoA; 2MBCAD: 2-methyl-branched chain acyl-CoA dehydrogenase; IVD: Isovaleryl-CoA dehydrogenase; 3-OH-VPA-CoA: 3-hydroxyl-valproyl-VPA; EH: 2-enoyl-CoA hydratase; 3-oxo-VPA-CoA: 3-keto-valproyl-CoA; HADH: hydroxyacyl-CoA dehydrogenase; MHBD: 2-methyl-3-hydroxybutyryl-CoA dehydrogenase; C3-CoA: propionyl-CoA; and C5-CoA: pentanoyl-CoA.

Valproic acid (VPA, 2-propylpentanoic acid), a branched-chain fatty acid, is originally extracted from *Valeriana officinalis*. VPA is commonly used in people with epilepsy because it is effective and can be administered orally, intravenously, or rectally. The oral bioavailability of VPA is more than 80%. Clinically, it is puzzling that the doses of VPA in the treatment of patients with epilepsy are variable and the toxicities of the drug are poorly correlated with VPA serum concentrations [101]. Studies showed that VPA has a very high protein binding (≥90%) in the plasma and few unchanged VPA (<3%) appears in the urine [102], suggesting a very complicated biotransformation of VPA in humans (Figure 4). First, 30%–50% of VPA may be metabolized via glucuronization by UGTs, including UGT1A3, UGT1A4, UGT1A6, UGT1A8, UGT1A9, UGT1A10, UGT2B7, and UGT2B15. End products are mostly excreted in the bile and urine. However, VPA can directly inhibit the activity of UGT1A4, and UGT2B7 [103,104].

Second, 30% of VPA metabolism occurs via β-oxidation in the mitochondria. VPA as a medium chain fatty acid is able to enter the mitochondrial matrix and is turned into valproyl-CoA (VPA-CoA) by medium-chain acyl-CoA synthase (EC 6.2.1.2) [105]. VPA-CoA is converted into VPA-dephospho-CoA and 2-propyl-valproyl-CoA (2-ene-VPA-CoA) by the phosphatase 2-methyl-branched chain acyl-CoA dehydrogenase (2MBCAD) and Isovaleryl-CoA dehydrogenase (IVD), respectively [106,107]. 3-hydroxyl-valproyl-VPA (3-OH-VPA-CoA) is generated from 2-ene-VPA-CoA through 2-enoyl-CoA hydratase (EH). 3-OH-VPA-CoA is converted into 3-keto-valproyl-CoA (3-oxo-VPA-CoA) or propionyl-CoA (C3-CoA) and pentanoyl-CoA (C5-CoA) by the action of 2-methyl-3-hydroxybutyryl-CoA dehydrogenase (MHBD) [108,109] or hydroxyacyl-CoA dehydrogenase (HADH) [105,108]. 3-oxo-VPA CoA is metabolized by the glutathione (GSH) into thiols [110]. 4-ene-VPA CoA, which is generated by the metabolism of VPA through 4-ene-VPA-CoA ester, is converted into 2,4-diene-VPA-CoA ester through 2MBCAD [110,111]. 2,4-diene-VPA-CoA and 4-ene-VPA-CoA are turned into thiols by GSH [110].

Third, 10% of VPA is biotransformed through CYP450-mediated oxidation. CYP2A6 is partially connected to the generation of 3-OH-VPA [112]. CYP2A6, CYP2C9, and CYP2B6 are involved in the VPA metabolism to generate 4-ene-VPA, 4-OH-VPA, and 5-OH-VPA [113]. Interestingly, VPA can inhibit CYP2C9, CYP2C19, and CYP3A4, but not CYP1A2, CYP2D6, or CYP2E1 [103,104]. VPA may undergo β-oxidation or glucuronidation when the doses are below or in therapeutic range [114]. This may explain why different doses of VPA cause distinct responses.

VPA affects the GABAergic system, inhibits α-ketoglutarate dehydrogenase (αKGD), GABA transaminase (GABA-T), and succinate semialdehyde dehydrogenase (SSD), and enhances glutamate decarboxylase (GAD) to elevate GABA levels in plasma and in several brain regions. Consequently, VPA may affect cerebral metabolism, activate GABA receptors to block sodium channels, and modulate calcium and potassium conductance and dopaminergic and serotoninergic transmission [115,116]. These mechanisms make VPA a multi-functional medication for absence, partial, and tonic-clonic seizures, bipolar disorder, depression, migraine, personality disorders or mental retardation, dementia and cognitive problems, and a potential chemotherapeutic agent [116]. Moreover, VPA can inhibit histone deacetylase (HDAC), which is a crucial factor in the pathogenesis of cancer and transcriptional regulation [117,118]. VPA is currently under investigation to be an adjunctive therapeutic option in neurodegenerative diseases, HIV, and cancers. Nausea, vomiting, abdominal cramps, diarrhea, weight gain, impaired coagulation, and neutropenia are the most common side effects of VPA. Hepatotoxicity, pancreatitis, teratogenicity, and endocrine disturbance, such as menstrual abnormalities, increased total testosterone levels, teratogenicity, obesity, and polycystic ovary syndrome (PCOS) may be associated with VPA. Hepatotoxicity is one of the most serious complications in the use of VPA. Although mitochondrial dysfunction and abnormal fatty acid metabolism have been proposed for the causes of VPA associated hepatotoxicity [119], the exact mechanisms are still unknown.

Fetal valproate syndrome is characterized by orofacial clefts, congenital heart disease, neural tube defects, limb defects, genitourinary defects, and craniosynostosis. VPA may affect limb and organ morphogenesis, suggesting a significant effect on bone growth and metabolism [120]. Long-term use of VPA may increase the risks of bone loss [19]. In vitro studies, our study [121] and others [122,123,124,125] showed that VPA may directly affect bone growth. VPA may have neuroprotective and anti-tumor activities through the modulation of epigenetic mechanisms [126,127,128]. VPA within therapeutic concentrations effectively inhibits histone deacetylases (HDACs). HDACs are enzymes crucial for the control of histone acetylation status and for the epigenetic regulation of gene activation involved in the modulation of cell growth, differentiation, and apoptosis [129,130]. VPA may cause short stature by directly inhibiting cell growth and proliferation through activation of apoptosis by hyperacetylation of histone tails and chromatin. In addition, serious side effects, teratogenesis, liver toxicity, and associated bone diseases have prompted the search for a newer generation of AEDs to provide better efficacy and fewer side effects (Table 1).

## 2. New Generation AEDs

### 2.1. Levetiracetam (LEV)

LEV ((S)-α-ethyl-2-oxo-1-pyrrolidine acetamide) was discovered through screening for effective AEDs in audiogenic seizure mice [131]. The chemical structure of LEV is the α-ethyl analog of piracetam and is unrelated to other AEDs [132]. LEV is a safe and well-tolerated new AED and no significant drug interactions were noted between LEV and concomitant medications because of lower protein binding and no involvement of hepatic CYP isozymes [131,132]. LEV is rapidly absorbed in the digestive tract and mainly excreted in urine. Approximately 1/3 of an administered dose of LEV was metabolized and 2/3 was excreted in urine in unchanged form [133]. The major pathway involves hydrolysis through the type B esterases primarily in the liver and blood [134] to generate (2*S*)-2-(2-oxopyrrolidin-1-y butanoic acid and two minor metabolites without significant pharmacological activities [135].

Pharmacologically, LEV effectively reduces partial seizures, intractable partial seizures, and patients with other medical conditions by several proposed mechanisms, including: (1) targeting synaptic vesicle protein 2A (SV2A), which is associated with vesicle neurotransmitter exocytosis; (2) negative modulation of neuron-associated GABA- and glycine-gated currents; (3) inhibiting voltage-gated calcium channels or reducing voltage-operated potassium currents [136,137,138,139]. Low-dose LEV was found to impair longitudinal skeletal growth and increase the risk of fractures in immature rats [140]. LEV was found to affect serum estradiol levels, suggesting that young and female individuals might be at risk of fractures with long-term use of LEV [141]. However, other reports [142,143] did not observe this effect. No reports are available regarding hydrolytic metabolites of LEV on bone diseases (Table 1).

### 2.2. Oxcarbazepine (OXC)

OXC (10,11-dihydro-10-oxo-5H-dibenz(b,f)azepine-5-carboxamide) has been designed via structural variation of CBZ [144]. After oral administration, metabolites of OXC in urine included MHD and two diastereoisomeric *O*-glucuronides (79%), unchanged OXC, OXC’s sulfate and glucuronide conjugates (13%), the *cis*- and *trans*-isomers of 10,11-dihydro-10,11-dihydroxy-carbamazepine (approximately 4%), and a phenolic derivative of MHD (less than 1%) [145]. Orally-administered OXC is rapidly metabolized to form the 10,11-dihydro-10-hydroxy-carbazepine (monohydroxy derivative, MHD) through cytosolic arylketone reductases. MHD is dissolved in water and a biologically active metabolite [144]. Therefore, MHD is as potent an anti-epileptic drug as OXC. MHD has two enantiomers: *S* enantiomers of MHD [(*S*)-MHD] (accounts for 80%) and *R* enantiomers of MHD [(*R*)-MHD] (accounts for 20%) [146]. The antiepileptic efficacy and tolerability of (*R*)-MHD and (*S*)-MHD is similar [147].

OXC, like CBZ, specifically inhibits voltage-dependent sodium [148], potassium [149], and calcium channels [150]. Although the efficacy of these two medications is similar, the safety of OXC is superior. Therefore, the FDA approved OXC as adjunctive therapy or monotherapy for children and adults with partial seizures. Hyponatremia is the main adverse effect of OXC [151]. Decreased BMD, altered levels of 25OHD [152,153], and bone turnover biomarkers such as PTH and bALP [152] were reported in patients with long-term OXC use [153,154]. However, our previous study [121] and others [155] did not discover any significant hypocalcemia or growth retardation in pediatric patients receiving OXC, and OXC did not significantly impair the proliferation of growth plate chondrocytes in an in vitro experiment [121]. Our recent study showed when patients with epilepsy took OXC and/or VPA for one year, their growth velocity was significantly decreased through affected bone resorption [156]. The use of VPA and/or OXC therapy affecting bone metabolism deserves further investigation (Table 1).

### 2.3. Lamotrigine (LTG)

LTG (6-(2,3-dichlorophenyl)-1,2,4-triazine-3,5-diamine) is rapidly and completely absorbed after oral administration. The oral bioavailability of LTG is 98%. The blood level of LTG is 1.4 to 4.8 h [157]. Metabolite identification studies demonstrate that *N*-2 glucuronide, *N*-5 glucuronide, *N*-2 methyl, and *N*-2 oxide are the main metabolites of LTG [158]. Most of these metabolites are non-active. LTG is eliminated via glucuronidation—mainly through UGT1A4, UGT2B7, and UGT1A1 [159]. LTG generally does not interfere with drug metabolizing enzymes. More frequent dosing and higher doses may be needed when co-administered with AEDs, such as PB, PT, CBZ, and OXC because these AEDs may enhance LTG clearance and decrease its plasma concentration by activating the glucuronidation pathway. On the contrary, co-administration of VPA may raise LTG plasma concentration as much as two-fold by inhibiting LTG clearance. Therefore, the recommended maintenance dose of LTG should be two-fold lower if LTG is co-administered with VPA. However, newer AEDs rarely affect LTG clearance [103].

LTG acts on pre-synaptical voltage-sensitive sodium channels. LTG blocks N- and P/Q/R-type calcium channels. These blocking effects and others may stabilize neuronal membrane potential [160]. LTG can abolish the repetitive firing in mouse spinal cord neurons in vitro. For these mechanisms, LTG is effective as a monotherapy or polytherapy for primary or secondarily generalized clonic-tonic seizures and simple or complex partial seizures. Additionally, LTG can be used as an adjuvant therapy in typical or atypical absence seizures, infantile spasms, juvenile myoclonic epilepsy, Lennox-Gastaut syndrome (LGS), and myoclonic seizures [161]. The antiepileptic effect of LTG is similar to that of PT and CBZ, but LTG are multi-functional when compared with these two drugs. LTG may inhibit the release of glutamate in the ventral part of the striatum and limbic areas, leading to the mood stabilization effect [161]. Headache, dizziness, sedation, nausea, insomnia, diplopia, and ataxia are common problems in patients taking this medication. The incidence of rash in the use of LTG is approximately 0.1% in all cases. The rash can vary from transient mild rash to fatal SJS. Children generally tend to have skin rashes more than adults. Adverse effects of LTG on bone, including bone loss [19], disturbed growth in children, impaired BMD, and elevated bone turnover markers have been reported [162] while our [121] and other [163,164,165] results were contradictory (Table 1).

### 2.4. Topiramate (TPM)

TPM (2,3:4,5-Bis-*O*-(1-methylethylidene)-β-d-fructopyranose sulfamate) is a derivative of monosaccharide d-fructose. TPM is rapidly and completely absorbed after oral administration and concomitant food intake does not affect the metabolism of TPM. The peak blood level of TPM is 1.4–4.3 h [166]. The protein binding of TPM in humans range 3%–4% [166]. An estimated 85% of an administered dose of TPM was predominantly excreted in urine as unchanged form [167]. The *t*_1/2_ of TPM is 19–25 h and is decreased by co-administration of EIAEDs such as CBZ and PT [166,167,168]. The remainder (15%) is metabolized through hydrolysis, hydroxylation, and glucuronidation. Six metabolites of TPM were detected in human urine without significant clinical activity [168]. TPM can partially inhibit CYP2C19 [169].

Pharmacologically, TPM affects GABAergic activity, inhibits voltage-sensitive sodium channels, calcium channels, and kainite/α-amino-3-hydroxy-5-methyllisoxazole-4-proprionic acid (AMPA)-type glutamate receptors, and blocks kinases to activate these channels [170]. All of these mechanisms not only make TPM approved as adjunctive therapy for adults and pediatric patients ages 2–16 years with primary generalized clonic-tonic seizures, partial seizures or LGS [171], but also contribute to a wide spectrum, including prophylaxis of migraines, alleviation of neuropathic pain, alcoholism, obesity, depression, bipolar disorder, and post-traumatic stress disorder [172]. Somnolence, nystagmus, hypo- or anhydrosis, paresthesia, poor concentration and word finding, weight loss, and decreased appetite, were the common complaints when using TPM. TPM may cause metabolic acidosis and nephrolithiasis through the inhibition of the carbonic anhydrase. The acid-base imbalance may accelerate osteopathy [173,174]. PTH secretion may be reduced after exposure to TPM, disrupting the balance between calcium resorption, the synthesis of 1,25(OH)2D, and the activities of osteoclasts [175]. In addition, TPM is a carbonic anhydrase inhibitor that may inhibit PTH-induced bone resorption, resulting in hypocalcemia. However, patients receiving TPM in our study did not show significant hypocalcemia or growth retardation [121]. More human studies may clarify these conflicting results (Table 1).

### 2.5. Gabapentin (GP)

GP (1-(aminomethyl) cyclohexane acetic acid), structurally-related to GABA, was originally developed to treat spasticity [176]. GP is absorbed in the gastrointestinal tract. The GP concentrations in CSF and brain are 20% and 80% of the concentrations in plasma, respectively [177,178]. GB can bind to voltage-dependent calcium channels containing the α_2_δ subunit to attenuate their activities [179,180]. GB does not bind to GABA_A_ or GABA_B_ receptors, nor does it disturb GABA uptake or metabolism, but can increase the concentration of GABA to reduce firing inputs [181] and enhance GABA responses in neuronal tissues [182]. For its high lipid solubility and structural uniqueness, GP can freely cross the blood-brain barrier, promptly elevate brain GABA, and presumably offer partial protection against further seizures within hours of the first dose [183]. GB inhibits neuronal calcium influx to reduce the release of mono-amine neurotransmitters, including glutamate, noradrenaline, and serotonin [184], causing decreased AMPA receptor activation in the brain. GB can bind to presynaptic NMDA receptors with inhibitory effects [185]. Due to these mechanisms that neither induce nor suppress hepatic microsomal enzymes [186], low level of protein binding [187], and renal excretion with an unchanged GB form in urine [178], GB is less likely to interact with other AEDs and is approved in persons over three years of age as an adjunctive AED for partial seizures with or without secondary generalization. In addition, GB can inhibit the descending noradrenergic system, leading to anti-hyperalgesic and anti-allodynic effects [188]. GB is effective in the treatment of a variety of pains including headaches, inflammatory pain, central pain, diabetic neuropathy, post-herpetic neuralgia, HIV-related neuropathy, trigeminal neuralgia, malignant pain, and postoperative pain management [176].

Sexual dysfunction, weight gain, dizziness, somnolene, and fatigue, but no serious idiosyncratic reactions or toxicities, have been reported [189,190]. Long-term GP therapy may increase the risks of fracture [42] and bone loss [19], suggesting that GP may have adverse effects on bone health (Table 1).

### 2.6. Vigabatrin (VB)

VB (4-amino-5-hexenoic acid) is a GABA-aminotransferase inhibitor to antagonize the GABA degradation in synapses [191]. VB is rapidly absorbed in small intestines and widely distributed throughout the body [192]. However, hepatic dysfunction has no impact on VB dosing because VB is predominantly excreted unchanged in the urine [192]. Lower doses are necessary in patients with renal dysfunction (creatinine clearance less than 80 mL/min). Younger subjects may demand a higher dose because their clearance is higher [104]. VB mainly relies on renal elimination and it does not need binding plasma proteins [192] or metabolism [193]. When patients with epilepsy were co-treated VB with other AEDs, VB might cause a significant increase in plasma clearance of CBZ [194] and decrease in the serum PT concentration [195]. VB is effective in the treatment of pediatric patients with infantile spasms, infantile spasms secondary to tuberous sclerosis, refractory complex partial seizures, and adult patients with LGS [196]. Patients treated with VB frequently complain of headache, ataxia, dizziness, tremors, fatigue, hyperactivity, and weight gain. Patients with myoclonic seizures should not use VB as it may aggravate this sort of seizure. Patients receiving VB should routinely undergo ophthalmological examinations because VB may damage the visual field. There was a study that enrolled patients with epilepsy receiving AEDs [197] and the study could not make a conclusion regarding the negative effects of VB on human bone metabolism because of limited subjects; however, immature rats treated with VB were found to have decreased body mass gain and inhibited compact bone growth [198]. Therefore, VB should be used cautiously in children, and the bone condition of pediatric patients should be closely monitored (Table 1).

## 3. Conclusions

AEDs are widely used for seizure treatment. However, abnormalities in bone and mineral metabolism have been frequently reported in individuals receiving EIAEDs because EIAEDs may cause hypocalcemia through triggering the catabolism of vitamin D. In vitro studies demonstrated that PB induces cultured human hepatocytes to increase the mRNA of CYP2C9, CYP2C19 [197], CYP2B6, and 3A4 [199]. Another in vitro study showed that CYP1A2, CYP2B6, and CYP3A4 were significantly induced by OXC and CBZ in HepaRG cells and human hepatocytes [200]. However, a systemic review analyzed 13 observational studies representing 68,973 patients with epilepsy. In all EIAED users, five studies illustrated decreased BMD; five studies demonstrated irrelevance to BMD; two studies reported increased incidence of fracture, and one study showed nothing to do with the incidence of fracture [201]. This finding led to no conclusion regarding the relationship between EIAEDs and bone metabolism. Additionally, it was reported that vitamin D deficiency was parallel to the low BMD in epilepsy patients on AEDs [19]. Numerous studies have shown that serum 25-hydroxyvitamin D levels are not significantly different between groups of subjects treated with either EIAEDs or NEIAEDs [60,202]. Moreover, calcium and vitamin D supplementation did not influence the prevalence of fractures in a retrospective study enrolling over 3000 patients with AEDs [203]. Taken together, these results raise public concerns on the bone growth or other medical conditions of children with epilepsy taking AEDs. So far, several newer-generation AEDs, including fosphenytoin, zonisamide, lacosamide, perampanel, eslicarbazepine, felbamate, ezogabine/retigabine, stiripentol, tiagabine, and rufinamide, have been designed and marketed [204]. Most of them have broader spectrums, fewer drug interactions, better tolerance, and minimal side effects, including bone diseases [205]. Timely withdrawal of AEDs and proper use of a new medication may avoid serious disabilities in users. In addition, supplementation of calcium and vitamin D are still recommended to epileptic patients on AEDs even though the effects of supplementation on AED-related osteopathy are controversial [206].

## Figures and Tables

**Table 1:** Review of literature regarding each anti-epileptic drug (AED) on the bone metabolism. Literature was classified into in vitro, in vivo, pediatric, adult, and animal group according to the study design. Abbreviation: BZD: benzodiazepines; CBZ: carbamazepine; PT: phenytoin; PB: phenobarbital; VPA: valproic acid; LEV: levetiracetam; OXC: oxcarbazepine; LTG: lamotrigine; TPM: topiramate; GP: gabapentin; VB: vigabatrin.

Drug	Study Design				
	In Vitro	In Vivo	Pediatric	Adult	Animal
BZD	48	24, 41, 42, 43, 44, 46, 47, 49	21, 44	24, 41, 42, 43, 46, 47, 79	
CBZ	77	19, 42, 58, 29, 60, 61, 79, 81, 164	58, 60, 194	42, 59, 60, 61, 79, 81, 164, 194	
PT	65, 66, 67, 77, 78, 79, 80, 82	42, 79, 80, 81, 86, 164		19, 42, 79, 80, 81, 86, 164, 195	
PB	100	19, 42, 45, 79, 81, 100	100	19, 42, 45, 79, 81, 100	100
VPA	121, 125	19, 121, 122, 123, 124, 164	121, 122, 124	123, 164	
LEV		140, 142, 143	142	142, 143	140
OXC	121	152, 153, 154, 156	153, 154, 155, 156	152, 155	
LTG	121	19, 121, 162, 163, 164, 165	121, 162, 164, 165	19, 163, 164	
TPM	121, 173	121, 174, 175		121, 174	121
GBP		19, 42		19, 42	
VGB	198	194, 195	194, 195	194, 195	
